# Attention Biases to Threat in Infants and Parents: Links to Parental and Infant Anxiety Dispositions

**DOI:** 10.1007/s10802-021-00848-3

**Published:** 2021-09-28

**Authors:** Evin Aktar, Cosima A. Nimphy, Mariska E. Kret, Koraly Pérez-Edgar, Maartje E. J. Raijmakers, Susan M. Bögels

**Affiliations:** 1 Department of Psychology, Clinical Psychology Unit, Wassenaarseweg 52, 2333AK Leiden, Netherlands; 2 Leiden Institute for Brain and Cognition (LIBC), Leiden University, Leiden, Netherlands; 3 Department of Child Development and Education, University of Amsterdam, Amsterdam, Netherlands; 4 Department of Psychology, Cognitive Psychology Unit, Leiden University, Leiden, Netherlands; 5 Department of Psychology, Child Study Center, The Pennsylvania State University, Pennsylvania, USA; 6 Department of Psychology, Developmental Psychology, University of Amsterdam, Amsterdam, Netherlands; 7 Department of Educational Studies, Vrije Universiteit Amsterdam, Amsterdam, Netherlands

**Keywords:** Attention, Emotion, Infancy, Parental anxiety, Parental stress, Temperament

## Abstract

Parent-to-child transmission of information processing biases to threat is a potential causal mechanism in the family aggregation of anxiety symptoms and traits. This study is the first to investigate the link between infants’ and parents’ attention bias to dynamic threat-relevant (versus happy) emotional expressions. Moreover, the associations between infant attention and anxiety dispositions in infants and parents were explored. Using a cross-sectional design, we tested 211 infants in three age groups: 5-to-7-month-olds (*n* = 71), 11-to-13-month-olds (*n* = 73), and 17-to-19-month-olds (*n* = 67), and 216 parents (153 mothers). Infant and parental dwell times to angry and fearful versus happy facial expressions were measured via eye-tracking. The parents also reported on their anxiety and stress. Ratings of infant temperamental fear and distress were averaged across both parents. Parents and infants tended to show an attention bias for fearful faces with marginally longer dwell times to fearful versus happy faces. Parents dwelled longer on angry versus happy faces, whereas infants showed an avoidant pattern with longer dwell times to happy versus angry expressions. There was a significant positive association between infant and parent attention to emotional expressions. Parental anxiety dispositions were not related to their own or their infant’s attention bias. No significant link emerged between infants’ temperament and attention bias. We conclude that an association between parental and infant attention may already be evident in the early years of life, whereas a link between anxiety dispositions and attention biases may not hold in community samples.

**Supplementary Information:**

The online version contains supplementary material available at 10.1007/s10802-021-00848-3.

Anxiety disorders are a highly prevalent cluster of mental illness (Remes et al., 2016) that runs in families (Beidel & Turner, 1997; Creswell & Waite, 2015; Eley et al., 2015; Gar et al., 2005; Hudson et al., 2011). Children of parents with an anxiety disorder are at increased risk for also developing an anxiety disorder. This increased risk is not simply due to inherited biological and temperamental dispositions for anxiety (e.g., Bolton et al., 2006; Dilalla et al., 1994; Lauet al., 2006; Robinson et al., 1992). Rather, children of anxious parents repeatedly witness their parents experience and express anxiety in response to stimuli in the environment that they deem threatening (e.g., Aktar et al., 2013). Thus, the intergenerational transmission of anxiety is shaped by a complex and dynamic interplay between genetic and environmental mechanisms (Creswell & Waite, 2015). Currently, little is known regarding the specific mechanisms that underlie social and behavioral mechanisms of intergenerational anxiety transmission.

Several information-processing theories suggest that cognitive processes, namely attention, memory, and interpretation biases to threat, play a fundamental role in the development, maintenance (Beck & Clark, 1997; Eysenck, 1992; Mathews & MacLeod, 2002; Mogg & Bradley, 1998), and parent-to-child transmission of anxiety (Creswell et al., 2010; Field & Lester, 2010). These theories propose that anxious individuals display biased cognitive processing of stimuli that subjectively signal threat. Among this cascade of cognitive processes is selective attention to threatening information, also known as attention bias to threat (Beck & Clark, 1997; Eysenck, 1992; Mog & Bradley, 1998; Mathews & MacLeod, 2002). Specifically, information-processing theories suggest that anxious individuals, compared to non-anxious individuals, tend to prioritize the processing of threat-relevant information over non-threat-relevant information. This argument is supported by empirical evidence noting an attention bias to threat-related information in anxious adults (Bar-Haim et al., 2007; van Bockstaele et al., 2014) and, more recently, in anxious children (Abend et al., 2018), as compared to non-anxious adults or children. Despite these commonalities in the anxiety-attention bias link in both childhood and adulthood, the role of attention biases to threat in the familial transmission of anxiety remains unknown.

Evidence supports a bidirectional causal link between attention biases and anxiety in anxious children and adults (Abend et al., 2018; van Bockstaele et al., 2014). This process builds on normative developmental mechanisms as a growing body of evidence from infant studies finds that attention biases to threat emerge as part of typical development between the 5th and 7th month of life (for a review, see Leppänen & Nelson, 2012). For example, in a study investigating infant attention to threat-relevant facial expressions, Leppänen and colleagues (2018) found that 7-, 12- and 36-month-old infants take longer to disengage from fearful as compared to happy faces, and 36-month-olds also take longer to disengage from angry as compared to happy faces. Other studies reported that 7-month-old infants spend longer times looking at fearful than happy faces (Nelson & Dolgin, 1985) and that 8-to-14-month-olds orient faster towards angry versus happy facial expressions (LoBue & DeLoache, 2010). Taken together, these findings suggest that an attention bias to threat is already observable for fearful expressions from 5-to-7 months onwards, and for angry expressions from 8-months onwards. Thus, attention biases towards social threat-related cues are present as part of typical development in infancy.

However, less is known regarding the potential links between these attention biases in infancy and anxiety dispositions in parents and infants. There is some evidence supporting the idea that infants who have been exposed to parental anxiety and fear responses show stronger attention biases to threat (see Burris et al., 2019 for a review). Morales and colleagues (2017) found that higher maternal anxiety was linked to stronger attention bias to angry faces in 4-to-24-month-old infants. Another study by Forssman et al., 2014 reported that 5-to-7-month-old infants of parents with higher levels of stress and depression showed a more pronounced attention bias for fearful faces. That is not to say that the data are uniform, as another study did not replicate the link between infant latency to disengage from threat-related expressions and parental anxiety in 5-to-36-month-olds (Leppänen et al., 2018). Thus, the evidence so far is inconclusive on whether a direct link is observed between parental anxiety dispositions and infant attention bias in community samples.

Studies examining the link between infant attention and infant anxiety risk have focused on infant fearful temperament as an early index of anxiety dispositions (Conejero & Rueda, 2018; Nakagawa & Sukigara, 2012). Infant temperamental profiles marked by fearful, distressed, and withdrawn responses to ambiguity and novelty are not only more common in the offspring of anxious parents (Rosenbaum et al., 2009), but are also the strongest predictor of childhood and adolescent anxiety (Clauss & Blackford, 2012). Moreover, it was proposed that infant predispositions for fearful temperaments, such as high behavioral inhibition (BI; White et al., 2017) and dysregulated fear (DF; Morales et al., 2015), may moderate the link of early attention biases to later anxiety in childhood.

There is also evidence providing support for a concurrent, cross-sectional link between infant attention to threat and fearful temperament. Nakagawa and Sukigara (2012) found that infants with a more negative temperament have more difficulty in disengaging from threatening faces, thus showing a higher threat-related bias at 12-months. Parallel findings were reported by Conejero and Rueda (2018) who observed that 9-to-12-month-olds with a more negative temperament take longer to disengage from fearful faces. Note, however, that Morales and colleagues (2017) did not replicate this link in 4- to 24-month-old infants. Taken together, the evidence from the few studies investigating early individual risk factors for child anxiety provide mixed findings with some support for significant links between infant and parental anxiety dispositions, and infant attention biases to threat.

Another cognitive-behavioral model suggests that the intergenerational transmission of information processing biases from parents to children can act as a potential mechanism for the familial aggregation of anxiety disorders (Creswell et al., 2010). The model builds on the argument that parents with anxiety disorders are more likely to detect threat in ambiguous stimuli or situations in their own, and their offspring’s environment. Parents’ selective processing of threat is then expected to induce selective attention for threat in the offspring, likely emerging from the parent repeatedly indicating to the child that the environment is threatening and modeling a threat response (Creswell et al., 2010). In other words, parental vigilance to threat and the accompanying expressions of anxiety in these situations may render the potentially threatening aspects of the environment more salient for their children, inducing a vigilance to threat, which, in turn, may lead to child anxiety. Based on this model, it can be suggested that the significant overlap between parental and offspring anxiety may be at least partially accounted for by shared attention biases in the processing of threat.

Currently, it remains unknown whether such an overlap in attention biases for parents and offspring is already observable in infancy, and whether it can at least partially account for the association between parental anxiety and child anxiety risk (Creswell et al., 2010). As Burris and colleagues (2019, p.12) point out: “To date, there is no research on how biased attention to threat in parents, parent psychopathology, and parental psychosocial stress together affect the developing biases of infants”. This study aims to bridge this gap in the literature by providing a first snapshot of the proposed associations between infant and parental attention biases to threat, and anxiety dispositions of infants and parents. We do so by relying on an initial cross-sectional design with three infant age groups from a community sample. For this study, infants visited the eye-tracking lab with one of their parents. We measured attention in the lab in infants and parents during free-viewing of dynamic threat-related (fearful and angry) versus positive dynamic facial expressions using eye-tracking. Parents reported on their anxiety and stress. When available, both parents of the participating infants were invited to complete online questionnaires that included infant temperamental fear and distress to limitations, which were used as an index of infant anxiety dispositions.

Our focus was on developmental windows corresponding to three important milestones in emotional development, at the end of their first (5–7 months), second (11–13 months), and third (17–19 months) half-year of life. As summarized above, a negativity bias in infant attention to threat-relevant expressions emerges between 5 and 7 months of life as part of typical development (Leppänen & Nelson, 2012; Vaish et al., 2008). At around the first year, emerging social referencing skills allow infants to incorporate others’ emotional and behavioral reactions to novel stimuli to accordingly determine their reactions (Feinman et al., 1992). Experience with full locomotion in the third half-year is an additional milestone known to change infants’ emotion processing (Campos et al., 2000; Clearfield, 2011), along with emerging linguistic understanding (Gervain & Mehler, 2010). To our knowledge, this study is the first to explore a potential moderation of the proposed interrelations between attention biases and anxiety dispositions in parents and infants, by age, in early development.

To summarize, we aimed to answer the following research questions (see Fig. 1 for an overview):

1. *Are parental anxiety dispositions related to parental attention bias to threat?* Based on earlier evidence in adult studies (Bar-Haim et al., 2007; van Bockstaele et al., 2014), we predicted that parents with higher levels of anxiety dispositions will show a stronger attention bias to threat-relevant emotional expressions (fearful and angry) as compared to happy expressions.
2. *Are infants’ anxious temperamental dispositions related to infants’ attention bias to threat?* Based on earlier evidence (Conejero & Rueda, 2018; Nakagawa & Sukigara, 2012), we expected a positive association between attention bias and temperamental anxiety dispositions among infants.
3. *Is there a relationship between parental and infant anxiety dispositions*? Based on earlier evidence family studies of anxiety (e.g., Bolton et al., 2006; Lau et al., 2006) we expected a significant positive association between anxiety predispositions of parents and infants.
4. *Is there a relationship between parental and infant attention bias to threat*? Based on information-processing models of anxiety (Beck & Clark, 1997; Eysenck, 1992; Mogg & Bradley, 1998; William et al., 1988) and the model on the intergenerational transmission of anxious information processing biases (Creswell et al., 2010), we expected to find a significant positive link in attention biases to threat-relevant expressions.
5. *How do parental anxiety dispositions together with parental attention bias, relate to infant attention bias to threat?* To understand how parental attention bias and parental anxiety dispositions together relate to infant attention bias (see Burris et al., 2019), we tested models that simultaneously included parental attention biases and anxiety dispositions as predictors of infant attention. We expected that high levels of attention bias and anxiety dispositions in parents would potentiate levels of attention bias in infants.

**Fig. 1:**
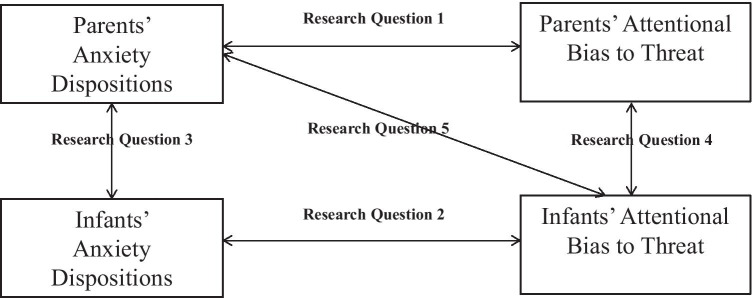
The overview of associations investigated in the current study

## Method

### Participants

For this study, Dutch infants visited the eye-tracking lab with one of their parents: the sample consisted of 211 infants and 216 parents (153 mothers). There were three age groups in the infant sample: 5-to-7-month-olds (*n* = 71, *Mage* = 6.11, *SD* = 0.51, range 5.00–7.50, 34 girls), 11-to-13-month-olds (*n* = 73, *Mage* = 12.11, *SD* = 0.59, range 10.70–12.90, 41 girls), and 17-to-19-month-olds (*n* = 67, *Mage* = 17.88, *SD* = 0.65, range 16.50–19.00, 34 girls). In 93.67% of the participating families, mothers reported being married to or living together with their partner. In addition to the parent who joined the experiment, the second parent, if available, was also invited to complete online questionnaires that included sociodemographic variables, as well as questionnaires capturing parental anxiety dispositions and infant temperament. Families were recruited from the community sample with invitation letters sent by the municipality to families with infants of the current age groups. The sociodemographic characteristics of the parents are presented in Table 1.

**Table 1:** Sociodemographic Characteristics of the Parents who Contributed to Attention Data

Age *M* (*SD,* range)Gender (% female, *N* female)Dutch origin % (*N*)	34.66 (4.38, 25–62)70.83% (153)74.07 % (160)
Highest Completed Educational level % (Frequency)	
Primary or secondary educationHigher professional educationScientific education	14.35 (31)23.15 (50)61.57 (133)
Professional level % (*N*)	
Predominantly manual labor or principal/main work requiring vocational trainingIndependent entrepreneurSalaried at LBO, MBO, or HBO levelSalaried employment requiring scientific training	8.34 (18)15.28 (33)37.04 (80)39.35 (85)
Monthly income *M* (*SD*, range)	
< 1000 euro1000 – 1999 euro2000 – 2999 euro3000 euro or more	14.35 (31)14.35 (31)22.69 (49)41.21 (89)

*N* sample size, *M* Mean, *SD* Standard deviation

From the initial sample of 251 families who visited the lab, the fixation data were not available from 14 infants and 14 parents due to child fussiness, tracking problems, software/equipment failure, or experimenter errors. Eye-tracking data from an additional 17 infants and 5 parents were removed due to missing data during data processing (for more information, see *Data Reduction*). Non-completers (*N* = 31, *Mage* = 9.91 months, *SD* = 4.38) were significantly younger than completers, *Mage* = 11.91 months, *SD* = 4.79), *t* (249) = 2.20, *p* = 0.029, but they did not differ from completers in gender, *p* = 0.123, or negative temperament, *p* = 0.758. The study was approved by the Ethics Review Board of the Department of Child Development and Education at the University of Amsterdam. Parents provided written informed consent for their own and their infants’ participation in the study.

## Materials and Procedure

### Stimuli

**Fig. 2:**
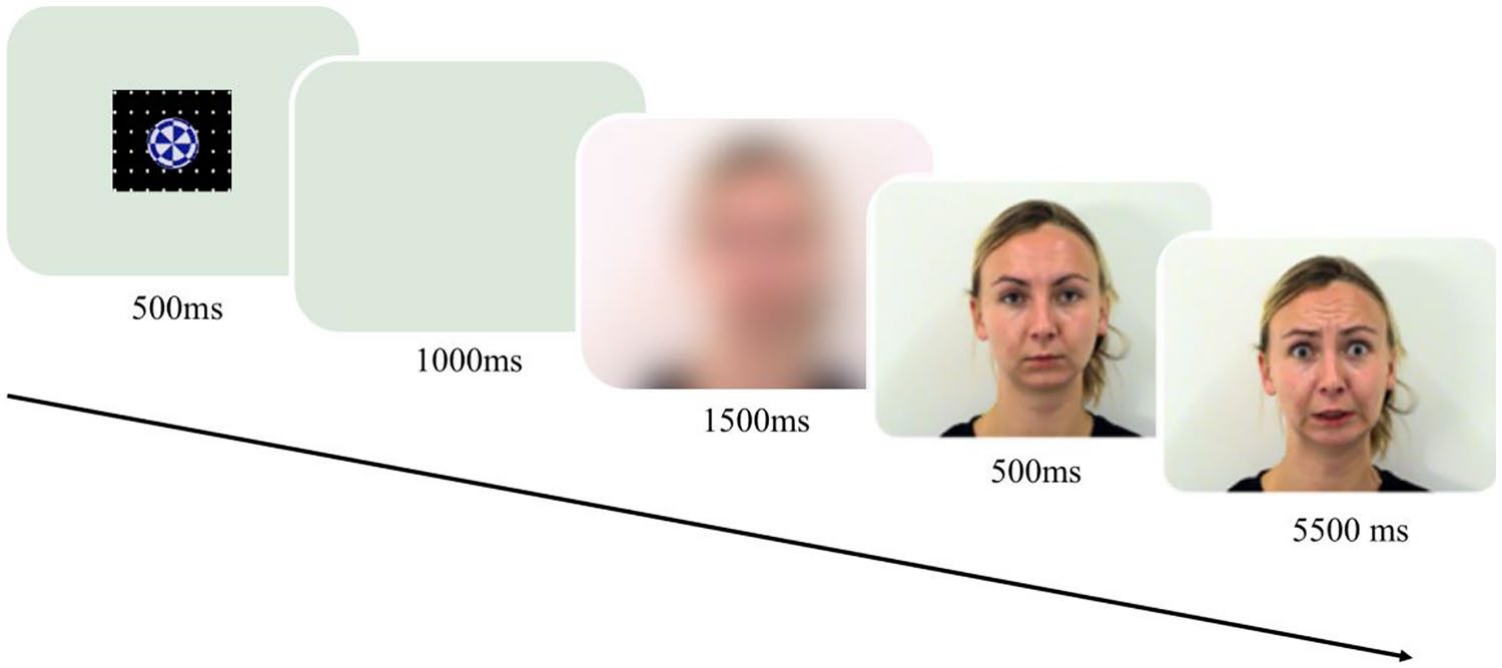
The time flow of a trial

A methodological limitation in many emotion processing studies in infants and adults concerns the use of static images of emotional expressions to measure attention biases (Bar-Haim et al., 2007; van Bockstaele et al., 2014; Vaish et al., 2008). Static images diverge from the everyday experience of emotions, which unfold in a temporally dynamic manner. To enhance ecological validity in the current experiment we made use of videos of facial expressions from 2 male and 2 female North European adults from ADFES (Van der Schalk et al., 2011) exhibiting neutral, happy, fearful, sad, and angry facial expressions. Each video started with a neutral expression that remained on the screen for 500 ms and was then followed by the onset of the expression, which reached the apex in 500 ms, and stayed at the apex for another 5 s (see Fig. 2). Our interest was in the comparison of attention to threat-relevant emotional expressions of fear and anger, as compared to happy facial expressions.

### Procedure

Dwell times were measured using an EyeLink® eye-tracker, with a sampling rate of 500 Hz. The experiment was presented on a 1280 × 1024 screen. Before the start of the experiment, participants’ gaze was calibrated and validated with a 5-point procedure. During the testing of the infant, the parent sat on a chair to the right of the infant. The parent was instructed to not intervene during the experiment unless the infant sought their attention or became fussy. The experimenter repeated the presentation of the attention-getter that preceded each trial in case the child was distracted. Following the testing of the infant, the parent was seated in front of the eye-tracking screen to complete the same task.

The experiment included four models presented in blocks of 5 dynamic emotions, repeated twice, for a total of 40 trials. Each trial started with a 500 ms attention-getter (e.g., circle expanding and contracting with sounds, see Fig. 2) continued with a 1000 ms of a blank screen, and with 1500 ms presentation of a blurred version of the face, followed by the dynamic stimuli (6000 ms). The first trial of each block was the neutral video of a given model, followed by the four dynamic emotional expressions of the same model in random order. All four models were presented in random order, before the presentation was repeated for a given model. The order by which the models were presented per block was randomly determined for infants and repeated for their parents.

Many infant emotion processing studies focus on positive emotional expressions as the non-threat-relevant reference stimuli (Vaish et al., 2008). In contrast, adult studies have predominantly used neutral faces as the non-threat-relevant reference (Bar-Haim et al., 2007; van Bockstaele et al., 2014). In the broader context of typical experience in infancy, exposure to positive expressions is the norm. As such, neutral faces are known to signal ambiguity. Indeed, non-contingent neutral faces are used as proxies for the parenting behaviors often seen among adults experiencing depression (Aktar, et al., 2017; Mesman et al., 2009). In the current experiment consisting of repeated presentation of dynamic facial expressions, the neutral faces perceptually differ from the rest of the stimuli not only by the absence of emotion, but also by the lack of dynamic facial movements (except for blinking). Given the differential perception of neutral faces in infancy and adulthood, and the relatively static nature of the neutral stimuli in the current experiment, we chose to use happy faces as the non-threat relevant emotional expression in the current study. Thus, our focus was on the comparison of threat-relevant negative (angry, fearful) vs. happy facial expressions.

## Data Reduction

The current study focused on dwell times as a behavioral measure of infant and parent attention. All available data from parents and infants were included in the analyses and were processed in a uniform way to increase comparability. Gaze data from parents and infants were extracted using EyeLink® Data Viewer (SR Research Ltd., Mississauga, Canada) using the default velocity, and acceleration-based algorithm. The dwell times to dynamic expressions were obtained by first summing the duration of the fixations to the face area (with a minimum duration of 100 ms) within each trial, and next by averaging the duration of the fixations across trials of a given emotion category. Data from 7 infants and 3 parents were missing following this reduction. Next, infants and parents who contributed less than 10 trials were removed from the analyses (*N* = 10 infants and 2 parents). At this stage, dwell times were available on average for 34.32 of 40 trials (*SD* = 6.75, *range:* 10–40) from infants and for 38.77 trials (*SD* = 3.63, *range:* 14–40) from parents. Following this data reduction, the trials with neutral and sad faces were excluded from further analysis.

### Questionnaires

In addition to the parent who visited the lab, the second parent, if available, was invited to complete online questionnaires that included sociodemographic variables, as well as questionnaires capturing parental anxiety dispositions and infant temperament. For analyses, we only used the self-report data from the parent who provided eye-tracking data. However, whenever available, we averaged temperament reports from both parents. Averaging helped minimize potential biases in parental perceptions of infant temperament, which are often related to their psychopathology and mood states (Kelley et al., 2017). This procedure also helped minimize effects due to shared method variance.

#### Parental anxiety dispositions***.*** 

We asked parents of the participating families to complete the Depression Anxiety Stress Scales (DASS; Lovibond & Lovibond, 1995), a 42-item questionnaire measuring depression anxiety and stress symptoms (14 items each) on a 4-point scale. For the current study, our interest was in the anxiety and stress scores of the parent who completed the eye-tracking experiment. These subscales tap into two distinct but related dimensions of anxiety dispositions that are relevant for the current study: the anxiety subscale captures physiological arousal and fearfulness, whereas the stress subscale assesses into negative affect component of generalized anxiety focusing on, for example, on tension and irritability (Brown et al., 1997). The distinction between general anxiety and anxious-arousal/panic dimensions may be however, relatively clearer in parents’ subjective experience than in their observable emotional displays. In other words, observable emotional displays corresponding to general anxiety and anxious-arousal may not be differentially perceived by infants. We, therefore, chose to incorporate both dimensions into our operationalization of parental anxiety dispositions. From 232 parents with attention data, the anxiety and/or stress scores were available from 216 parents. The internal consistency (Cronbach α) in this sample was 0.70 and 0.88 respectively for the anxiety and stress subscales. The correlation between the anxiety and stress scores was *r* (216) = 0.45, *p* < 0.001 (see Table 2 an overview of the descriptives and correlations between infant temperamental dispositions, visiting parents’ anxiety, and stress scores). The mean score of the visiting parent in each of the two subscales was averaged to obtain the final measure of parental anxiety dispositions.

**Table 2:** Intercorrelations between infant temperamental anxiety dispositions, and parents’ anxiety and stress

	*N*	*Range*		*M*	*SD*	2	3
1. Infant Temperamental Anxiety Dispositions	216	1.56–4.82		2.68	0.58	0.10	0.16*
2. Parental Anxiety	216	0—13		1.32	2.03		0.45**
3. Parental Stress	216	0—36		6.40	5.27		

*N* sample size, *M* Mean, *SD* Standard deviation, 1 Infant Temperamental Anxiety Dispositions, 2 Parental Anxiety, 3 Parental Stress

*p ≤ 0.05; ** *p* ≤ 0.01

#### Infant temperamental dispositions for anxiety. 

Both parents of participating children were invited to complete a shortened version of the Infant Behavior Questionnaire-Revised (IBQ-R; Gartstein & Rothbart, 2003), which included subscales measuring temperamental dimensions related to positive (e.g., smile and laughter) and negative (e.g., fear, sadness, and distress to limitations) emotions on a 7-point scale. For the current study, our interest was on the fear and distress to limitation subscales, each consisting of 16-items. Internal consistency (Cronbach α’s) for these subscales in this study was 0.80 and 0.82 for mothers’, and 0.80 and 0.81 for fathers’ ratings, respectively. The correlation between maternal ratings of distress to limitation and fear was *r* (195) = 0.41*, p* < 0.001. The correlation between paternal ratings of distress to limitation and fear was *r* (163) = 0.32, *p* < 0.001. To compute infant temperamental dispositions for anxiety, we first averaged each parent’s scores on these two dimensions. We obtained the final temperament scores by averaging maternal and paternal ratings of temperamental anxiety dispositions. When the scores were available from one parent only, these were used as final scores. From 220 infants with attention data, scores on infant temperament were available for 211 infants.

The intercorrelations between infant temperamental anxiety dispositions and parents’ anxiety dispositions are presented in Table 2. There was a positive association between infants’ temperament and parents’ stress *r* (216) = 0.16, *p* = 0.019, but not anxiety scores, *p* = 0.156. The correlation between the final scores on parents’ anxiety dispositions (the mean of anxiety and stress scores) and infant temperament was *r* (216) = 0.23, *p* = 0.001.

## Statistical Analyses

Statistical analyses were conducted using IBM SPSS Statistics for Mac, Version 25.0. We used multi-level regression models to account for the repeated structure of the dataset. The main outcome measure in the analysis of infants’ and parents’ attention biases was the mean dwell time per emotional expression. Emotion was initially randomized in the mixed models with infants’ and parents’ dwell times as the outcome variable, but was redundant, and therefore not randomized further in the analyses. Emotion was, therefore, included as a fixed effect with the happy faces as reference. We used maximum likelihood for estimation in all models. The intercept was randomized. The initial models included theoretically relevant interactions (specified per model below, and presented in the Supplement).

To reach the most concise final models, we removed the non-significant interactions one-by-one starting from higher-order interactions and higher *p*-values. 6-month-olds were the reference age group in infant analyses. Parental anxiety scores and infant anxious temperament scores were entered as continuous variables in the models. Scores from all the outcome and predictor variables were standardized in the analyses. Inspection of distributions indicated sufficient normality for the scores of infant dwell times and infant temperament (both skewness and kurtosis values were < 0.5). Parental dwell times and their anxiety disposition scores had relatively higher skewness and kurtosis levels (skewness and kurtosis were -2.45 and 6.28, and 1.70 and 3.97 respectively). The distribution of residuals was therefore further inspected for the models including these variables as predictor or outcome variables. These indicated sufficient normality (all residuals had skewness values < 1 and kurtosis values < 2.29).

## Results

1. *Are parental anxiety dispositions related to parental attention bias to threat?*
  We investigated the link between parental anxiety dispositions and parental attention biases to threat in a multi-level regression model (*N* = 216) with parental dwell times as the outcome variable, and the main effects of Emotion (angry and fear versus happy), parent gender, and parental anxiety dispositions (averaged anxiety and stress scores on the DASS) along with all the two-way and three-way interactions between these three variables as predictors. None of the tested interactions were significant in the initial model (Table S1, *p’*s > 0.086) reducing this model to the main effects model (see Table 3). This model revealed no significant association between parental anxiety and differential attention to threat-relevant (versus happy) faces, *p* = . 811. The main effect of parent gender was also not significant, *p* = 0.421, pointing to the lack of significant differences between mothers and fathers in this sample. The main effect of emotion was marginally significant in this model *F* (2, 338.05) = 2.57,* p* = 0.078. Estimates for differential attention to angry and fearful (versus happy) faces revealed that parents dwelled significantly longer on angry than happy faces, *β* = 0.05, *SE* = 0.02, *p* = 0.041, whereas there was only a trend toward longer dwell times on fearful versus happy faces, *β* = 0.04, *SE* = 0.02, *p* = 0.062. We conclude that an attention bias was especially observable for angry rather than fearful versus happy faces, whereas no association was found between self-report of anxiety dispositions and attention to threat-relevant versus happy expressions.
2. *Are infants’ anxious temperamental dispositions related to attention bias to threat?*
  Next, we investigated the link between infant’s dispositions for anxiety and attention biases to threat in a multi-level regression model (*N* = 211) with infant dwell times as the outcome variable, and the main effects of Emotion (angry, fear versus happy), age group (12 and 18-months vs. 6-month-olds), and infant temperamental anxiety dispositions (averaged ratings of parental report of fear and distress to limitations), and all the two-way and three-way interactions between these variables as predictors. None of the tested interactions were significant in the initial model (Table S2, *p’*s > 0.532) reducing this model to the main effects model (see Table 4). This model revealed no significant association between temperamental anxiety dispositions and differential attention to threat-relevant (versus happy) faces, *p* = 0.490. There was a significant main effect of emotion *F* (2, 330.34) = 13.44,* p* < 0.001. Infants dwelled marginally longer on fearful, *β* = 0.08, *SE* = 0.04, *p* = 0.057 than happy faces, like parents. In contrast, they dwelled shorter on angry, *β* = -0.14, *SE* = 0.04, *p* = 0.002, than happy faces. A significant main effect of age group, *F* (2, 210.94) = 8.20,* p* < 0.001, revealed overall longer dwell times to emotional expressions in 12-month-olds (*β* = 0.44, *SE* = 0.16, *p* = 0.006) and 18-month-olds (*β* = *0.6*4, *SE* = 0.16, *p* < 0.001) as compared to 6-month-olds irrespective of emotion, revealing the increasing span of attention with increasing dwell times in the first 18-months. The mean dwell times across emotion categories were 2518.48 ms (*N* = 70, *SD* = 1512.62) for the 6-month-olds, 3117.36 ms (*N* = 73, *SD* = 993.53) for 12-month-olds, and 3372.80 ms (*N* = 68, *SD* = 945.50) for 18-month-olds.
  Taken together, results reveal a marginally significant negativity bias in infant attention to fearful faces, whereas an avoidant pattern was observed when comparing angry to happy faces, with more attention to happy than angry faces. No significant links appeared between infant attention to threat-relevant faces and their temperamental anxiety dispositions.
3. *Is there a relationship between parental and infant anxiety dispositions?*
  We tested the association between parental anxiety dispositions and infant temperamental anxiety dispositions in a simple linear regression model with the infant anxious temperament as the outcome, and infant age group (12 and 18-month-olds vs. 6-month-olds), and parental anxiety dispositions as predictors, *N* = 196. The two-way interactions between age and parental anxiety dispositions were also tested in this model, but were not significant (*p’*s > 0.340 see Table S3), reducing the final model to the main effects (see Table 5). This model revealed a marginally significant link between parental and infant anxiety dispositions, *β* = 0.12, *SE* = 0.16,* p* = 0.061. A main effect of age group revealed significantly higher levels of anxious temperament in 12-month-olds *β* = 0.41, *SE* = 0.09,* p* < 0.001, and 18-month-olds *β* = 0.41, *SE* = 0.09,* p* < 0.001, as compared to 6-month-olds. Thus, findings reveal a trend for a relationship between parents’ own and their infant’s anxiety dispositions, and a significant increase in levels of anxious temperamental dispositions in infants in the first 18 months.
4. *Is there a relationship between parental and infant attention biases to threat*?
  Next, we explored the dependency between emotion processing of parent-infant dyads by investigating the association between infant and parental dwell times in multi-level models with infant average dwell times to separate emotions (averaged across trials per emotion category) as the outcome variable, and emotion and parental average dwell times as predictors. The initial model included infant age group and parent gender as well as all the two-way and three-way interactions between emotion, infant age group, parent gender, and dwell times as fixed predictors. None of the two and three-way interactions were significant in the initial model, see Table S4, *N* = 210 (*p’s* > 0.313), reducing the final model to the main effects model. This final model (presented in Table 6) revealed a significant positive association between parental and infant dwell times, *F* (1, 431.43) = 4.91,* β* = 0.11, *SE* = 0.05,* p* = 0.027. Thus, infants of parents who have longer dwell times on threat-relevant and happy expressions also showed longer dwell times on threat-relevant and happy emotional expressions. This link did not significantly differ as a function of emotion, infant age, or parent gender.
5. *How do parental attention bias together with parental anxiety, relate to infant attention bias to threat?*

**Table 3:** Multi-level regression of parents' dwell times on threat-relevant (versus happy) emotional expressions and parental anxiety dispositions (N = 216)

Fixed Effects	*Numerator df*	*Denominator df*	*F*	*p*			
Intercept	1	215.97	0.41	0.525			
Emotion (threat-relevant versus happy)	2	338.05	2.57	0.078			
Parent Gender (mother versus father)	1	215.96	0.65	0.421			
Parental Anxiety Dispositions	1	215.96	0.06	0.811			
Estimates of Fixed Effects							
Parameters	*ß*	*SE*	*df*	*t*	*p*	95% Confidence Intervals	
						Lower Bound	Upper Bound
Intercept	-0.04	0.11	222.37	-0.37	0.709	-0.26	0.18
Angry versus Happy	0.05	0.02	221.33	2.05	0.041	0.00	0.10
Fearful versus Happy	0.04	0.02	350.25	1.87	0.062	0.00	0.09
Mother (vs. Father)	0.11	0.13	215.96	0.81	0.421	-0.15	0.37
Parental Anxiety Dispositions	0.01	0.06	215.96	0.24	0.811	-0.10	0.13
Estimates of Covariance Parameters		*Estimate*	*SE*	*Wald Z*	*p*	Lower Bound	Upper Bound
Repeated Measures	AR1 diagonal	0.06	0.01	9.95	< 0.001	0.05	0.07
	AR1 rho	0.04	0.10	0.36	0.719	-0.16	0.23
Intercept [subject = ID]	Variance	0.75	0.07	10.10	< 0.001	0.62	0.91

*df* degrees of freedom, *F* F-value, *p* significance level, *ß* Beta, *SE* Standard Error, *t* t-value

**Table 4:** Multi-level regression of infant dwell times on threat-relevant (versus happy) emotional expressions, and infant anxiety dispositions (N = 211)

Fixed Effects	*Numerator df*	*Denominator df*	*F*	*p*			
Intercept	1	210.95	0.02	0.896			
Age group (12 and 18 versus 6-month-olds)	2	210.94	8.20	<0 .001			
Emotion (threat-relevant versus happy)	2	330.34	13.44	<0 .001			
Infant Anxiety Dispositions	1	210.94	0.48	0.490			
Estimates of Fixed Effects							
Parameter	*ß*	*SE*	*df*	*t*	*p*	95% *CI*	
						Lower Bound	Upper Bound
Intercept	-0.34	0.11	230.85	-2.93	0.004	-0.56	-0.11
12 versus 6-month-olds	0.44	0.16	210.94	2.79	< 0.006	0.13	0.75
18 versus 6-month-olds	0.64	0.16	210.94	4.01	< 0.001	0.33	0.96
Angry versus Happy	-0.13	0.04	210.28	-3.18	0.002	-0.22	-0.05
Fearful versus Happy	0.08	0.04	346.42	1.91	0.057	0.00	0.16
Infant Anxiety Dispositions	0.05	0.07	210.94	0.69	0.490	-0.09	0.18
Estimates of Covariance Parameters		*Estimate*	*SE*	*Wald Z*	*p*	Lower Bound	Upper Bound
Repeated Measures	AR1 diagonal	0.19	0.02	9.76	< 0.001	0.15	0.23
	AR1 rho	0.03	0.10	0.31	0.755	-0.17	0.23
Intercept [subject = ID]	Variance	0.69	0.08	9.17	< 0.001	0.56	0.86

*df* degrees of freedom, *F* F-value, *p* significance level, *ß* Beta, *SE* Standard Error, *t* t-value

**Table 5:** Linear regression of infant temperamental anxiety dispositions on parental anxiety dispositions (N = 196)

	*B*	*SE*	*ß*	*t*	*p*		95% Confidence Intervals	
						Lower Bound		Upper Bound
Intercept	2.28	0.08		29.95	< 0.001	2.13		2.43
12 versus 6-month-olds	0.50	0.09	0.41	5.43	< 0.001	0.32		0.68
18 versus 6-month-olds	0.50	0.09	0.41	5.39	< 0.001	0.31		0.68
Parental Anxiety Dispositions	0.30	0.16	0.12	1.89	0.061	-0.01		0.62

*B *unstandardized estimate, *SE* Standard Error, *ß* Beta, *t* t-value, *p* significance level

**Table 6:** Multi-level regression of infant dwell times on threat-relevant (versus happy) emotional expressions, parent gender, age group, and parental dwell times (N = 210)

Fixed Effects	*Numerator df*	*Denominator df*	*F*	*p*				
Intercept	1	209.47	0.31	0.577				
Parent Gender (mother versus father)	1	209.57	1.03	0.311				
Age group (12 and 18 versus 6-month-olds)	2	210.71	9.45	< 0.001				
Emotion (threat-relevant versus happy)	2	328.02	14.53	< 0.001				
Parental Dwell Times	1	431.43	4.91	0.027				
Estimates of Fixed Effects								
Parameter	*ß*	*SE*	*df*	*t*	*p*	95% Confidence Intervals		
						Lower Bound	Upper Bound	
Intercept	-0.25	0.13	225.20	-1.85	0.066	-0.51	0.02	
Mother (vs. Father)	-0.13	0.13	209.57	-1.02	0.311	-0.39	0.13	
12 versus 6-month-olds	0.44	0.15	209.48	2.96	0.003	0.15	0.73	
18 versus 6-month-olds	0.65	0.15	211.56	4.24	< 0.001	0.35	0.94	
Angry versus Happy	-0.13	0.04	211.03	-3.09	0.002	-0.22	-0.05	
Fearful versus Happy	0.09	0.04	344.62	2.22	0.027	0.01	0.18	
Parental Dwell Times	0.11	0.05	431.43	2.22	0.027	0.01	0.21	
Estimates of Covariance Parameters			*SE*	*Wald Z*	*p*	Lower Bound	Upper Bound	
Repeated Measures	AR1 diagonal	0.19	0.02	9.70	< 0.001	0.16	0.24	
	AR1 rho	0.04	0.10	0.35	0.730	-0.17	0.24	
Intercept [subject = ID]	Variance	0.70	0.08	9.14	< 0.001	0.57	0.87	

*df* degrees of freedom, *F* F-value, *p* = significance level, *ß* Beta, *SE* Standard Error, *t* t-value

Finally, we investigated individual differences explained by parental anxiety dispositions, as well as parental dwell times on infant dwell times in models that simultaneously included the main effects of emotion, parental anxiety dispositions, and dwell times, along with all the two and three-way interactions between these predictors, *N* = 195. None of the tested interactions were significant in the initial model, see Table S5, (*p*’s > 0.153), reducing the final model (presented in Table 7) to main effects. This model revealed no significant main effect of parental anxiety, *p* = 0.934, whereas the significant link between parent and infant dwell times remained significant *F* (1, 441.72) = 4.86, *β* = 0.12, *SE* = 0.06,* p* = 0.028. We conclude that the relationship between parent and infant attention to threat-relevant emotions holds independent of parental anxiety dispositions. In a final step, we explored whether a link exists between parental anxiety and infant attention before controlling for parental attention. Parental anxiety did not predict infant attention, neither alone *p* = 0.942, nor as a function of infant age, or parent gender, *p’s* > 0.239. We conclude that the link between parental anxiety dispositions and infant dwell times is not significant, regardless of any concordance in attention bias in parent-infant dyads.

**Table 7:** Multi-level regression of infant dwell times on threat-relevant (versus happy) emotional expressions, age group, and parental gender, dwell times and anxiety dispositions (N = 195)

Fixed Effects	*Numerator df*	*Denominator df*	*F*	*p*				
Intercept	1	194.79	0.57	0.453				
Parent Gender (mother versus father)	1	193.96	1.97	0.162				
Age group (12 and 18 versus 6-month-olds)	2	194.84	11.06	< 0.001				
Emotion (threat-relevant versus happy)	2	298.31	12.59	< 0.001				
Parental Dwell Times	1	441.72	4.86	0.028				
Parental Anxiety Dispositions	1	193.97	0.01	0.934				
Estimates of Fixed Effects								
Parameter	*ß*	*SE*	*df*	*t*	*p*	95% Confidence Intervals		
						Lower Bound	Upper Bound	
Intercept	-0.24	0.14	207.90	-1.76	0.080	-0.51	0.03	
Mother (vs. Father)	-0.19	0.14	193.96	-1.41	0.162	-0.46	0.08	
12 versus 6-month-olds	0.55	0.15	194.25	3.65	< 0.001	0.25	0.85	
18 versus 6-month-olds	0.67	0.15	195.70	4.42	< 0.001	0.37	0.97	
Angry versus Happy	-0.14	0.04	196.48	-3.12	0.002	-0.23	-0.05	
Fearful versus Happy	0.08	0.04	324.14	1.74	0.083	-0.01	0.16	
Parental Dwell Times	0.12	0.06	441.72	2.21	0.028	0.01	0.23	
Parental Anxiety Dispositions	0.01	0.06	193.97	0.08	0.934	-0.12	0.13	
Estimates of Covariance Parameters		*Estimate*	*SE*	*Wald Z*	*P*	Lower Bound	Upper Bound	
Repeated Measures	AR1 diagonal	0.195	0.022	8.916	< 0.001	0.156	0.243	
	AR1 rho	0.067	0.111	0.607	0.544	-0.15	0.28	
Intercept [subject = ID]	Variance	0.648	0.075	8.623	< 0.001	0.52	0.81	

*df* degrees of freedom, *F* F-value, *p* significance level, *ß* Beta, *SE* Standard Error, *t* t-value

## Discussion

This study aimed to investigate the links between parental and infant attention biases to threat, along with anxiety dispositions in these same infants and parents. The current findings reveal a significant positive association between attention to threat-relevant (versus happy) facial expressions in parents and infants, and a marginally significant positive link between parental and infant anxiety dispositions. In turn, there was no significant link between an individual’s attention biases and his or her anxiety dispositions. This held for both infants and parents. Parental anxiety dispositions were also not related to their infant’s attention biases to threat. Below we discuss each of these findings in more detail within the broader framework of information processing model of intergenerational anxiety transmission (Creswell et al., 2010)

Creswell and colleagues (2010) suggest that an anxious parent’s vigilance to potentially threatening stimuli or situations in their environment may trigger or potentiate selective attention for threat in the offspring, leading to child anxiety. Accordingly, the link between parental and offspring anxiety is proposed to be at least partially accounted for by shared attention biases in the processing of threat. The current study was a first preliminary investigation of the proposed overlap in attention and anxiety dispositions of parents and infants, in a cross-sectional, correlational design, without a formal mediation analysis. In line with family studies of anxiety (e.g., Bolton et al., 2006; Lau et al., 2006; Creswell & Waite, 2015), the findings revealed a trend for an association between parental and infant anxiety dispositions: Parents who reported higher levels of anxiety and stress had infants who showed higher levels of temperamental fear and distress. A methodological limitation in the current design concerns the use of parental reports for measuring both infant and parental anxiety dispositions, as a parent’s anxiety dispositions may bias their perception of child temperament (Kelley et al., 2017). We aimed to address this limitation by using reports of infant temperament from both parents when available. Our findings suggest that the relationship between parental traits and infant risk is evident, and stable, across the first 18 months of life. This is in line with the findings capturing shared genetic substrates that influence psychosocial functioning in both generations (Bolton et al., 2006; Lau et al., 2006). Needless to say, genetically-informed studies are needed in order to disentangle the genetic and environmental contributors to this overlap in typical development (Creswell & Waite, 2015). However, even within the limitations of the current within-family cross-sectional design, the use of multiple informants helps alleviate some of these concerns. We then build on this foundation to examine concordance in a putative functional mechanism for intra- and inter-individual patterns of anxiety.

To our knowledge, this is the first study to explore the potential overlap in infant and parental attention biases to threat-relevant versus happy emotional expressions as a mechanism explaining the relationship between infants’ and parents’ anxiety in a free-viewing experiment. In line with the proposal by Creswell and colleagues (2010), the findings revealed that parents of infants who showed more attention to emotional faces also showed more attention to emotional faces. Thus, this link was not specific to threat-relevant expressions, but also extended to happy faces. Despite this overall link in the attention of parents and infants, separate analyses of parent and infants reveal differences in their relative attention to threat-relevant faces: Although both parent and infants dwelled marginally longer on fearful than happy faces, attention to angry versus happy faces differed between infants and parents: Parents spent longer time fixating on angry expressions compared to happy faces, whereas a reverse pattern was observed for infants who spent longer time looking at happy than angry faces. Thus, in contrast to parents, infant negativity bias was specific to fear, whereas an avoidant response was observed for angry expressions.

There is some earlier evidence that suggests that an attention bias for fearful faces may precede angry faces in development, which may emerge sometime between eighth month and third year of life (Leppänen et al., 2018; LoBue & DeLoache, 2010). A bias for angry faces was not observed in the current study, which may suggest that our sample did not capture the appropriate developmental window. The avoidant attention pattern observed among infants in the current study may be related to the differences in the threat value of angry and fearful expressions or infant’s differential exposure to anger and fear in everyday situations. In the case of a fearful face, the signal often points to an indirect threat, as the individual is reflecting their response to an external stimulus as yet unknown to the infant (Adams & Klerck, 2003). As such, the signal is ambiguous and more computationally taxing as the infant must deduce the source of the emotional response. In contrast, when observing an angry face, the individual is the direct source of the threat. The direct threat value of angry faces may have triggered an avoidant reaction among infants in the current study. As an alternative, we can consider the prototypical pattern of faces infants are exposed to in their daily experience. The assumption is that in adaptive and supportive environments, infants are likely to have more direct experience with positive (happy) faces relative to anger and fear. Angry faces, in particular, may be rare in a community sample, leading to the pattern of responses noted here. While these different lines of reasoning need to be tested, the core finding of the current study suggests that, within the first 18 months of life, infants and parents display parallel patterns of attention, in line with proposed mechanisms of intergenerational transmission (Creswell et al., 2010).

Despite a significant overlap between parental and infant attention, as well as a marginally significant overlap in anxiety profiles, no empirical support was found for the idea that this overlap in attention can partially or fully account for the overlap in anxiety profiles in this community sample: no significant associations were found between attention biases and anxiety dispositions, in either infants or parents. Earlier evidence from adult studies has revealed a link between attention biases to threat and anxiety in adults with clinical or subclinical levels of anxiety (Bar-Haim et al., 2007; van Bockstaele et al., 2014). In contrast to earlier studies that noted a link between parental anxiety and infant attention biases to threat in infancy (Burris et al., 2019; Forssman et al., 2014; Morales et al., 2017), we failed to note a similar relation in the current study (before and after considering parental attention biases).

The lack of significant associations between parental anxiety and attention biases in either infants or parents may be explained by the fact that most of the participating parents reported little to mild levels of anxiety dispositions in this community sample. Out of 216 parents who contributed to the current study, only 4 parents reported moderate levels of anxiety, and only 8 parents reported moderate or higher levels of stress. Thus, in the current parent sample, there was more individual variation in the generalized anxiety symptoms and associated negative affect dimension, than in the physiological arousal and fearfulness dimension of anxiety, which may characterize rather subclinical and clinical levels of anxiety displays. Overall, these rates of psychopathology in our sample seem to be lower than rates reported in community samples (Bijl et al., 1998), thus non-anxious parents may have been more likely to join this infant study. The limited number of parents with moderate or clinical levels of anxiety dispositions may have limited our ability to detect any associations were they to exist across a fuller spectrum of anxiety (Bar-Haim et al., 2007).

We also failed to note a link between infant anxiety dispositions (a composite measure of temperamental fear and distress reported by both parents) and their attention biases. This finding is not consistent with earlier evidence suggesting that infants with a more negative temperament take longer to disengage from threat (Conejero & Rueda, 2018; Nakagawa & Sukigara, 2012), although Morales and colleagues (2017) found no significant link between temperamental negativity and disengagement from threat. Here, we may point to differences in the task at hand. The prior studies used an overlap task that captured the infant’s ability, and propensity, to disengage from a face when provided with an alternate stimulus in the periphery of the visual field. Here, the infants were provided with a single, albeit dynamic, face at a time. As such, we may have captured the infant’s pattern of processing of, and interest in, the face, as opposed to comparing competing targets for the infant’s limited attentional resources. Indeed, Rothbart and Posner (2006) note that temperamental variation may differ across associated components of attention, such as vigilance and disengagement. Future studies are needed to further specify the nature of this association and to answer whether the inconsistent findings are related to child or task-related factors.

Across the core analyses, we noted an absence of any age effects. Although the findings reveal higher levels of temperamental anxiety dispositions in 12 and 18-month-olds, this uptick did not moderate the links between parent-infant attention or anxiety dispositions. The longer dwell times observed in 12 and 18-month-olds may reflect the normative improvement in attentional control with age. This finding is also in line with an earlier study reporting an increase in attention (i.e., dwell time to angry faces) between 4 and 24 months of age (Pérez-Edgar et al., 2017). However, the hypothesized relations with individual and parental traits were not evident here.

Taken together, current findings provide support for an overlap between infant and parental attention biases to threat, and a more tenuous relationship between infant and parental anxiety dispositions. However, we found no empirical support for relations crossing constructs of interest (attention vs. anxiety) for either infants or parents. Thus, the suggestion we based on the model by Creswell and colleagues (2010) that concordance in anxiety dispositions would be at least partially be accounted for by attention biases to threat, does not seem to hold in this community sample. Future investigations of these associations should include clinically and sub-clinically anxious parents and their infants, and should test the model by Creswell et al. (2010) in formal mediation analyses.

## Clinical Implications

The current study constitutes the first evidence for a link between parental and offspring attention biases as early as infancy. Although there was a marginally significant link between parent and child anxiety dispositions, the parents in this community sample reported low levels of anxiety, precluding strong conclusions regarding the relations predicted by the Creswell et al. (2010) model for the intergenerational transmission of anxiety.

The significant link between infant and parental attention bias is likely to be adaptive in typical development in a window marked by infants increasingly exploring the world around them under their parent’s guidance (e.g., Koterba & Iverson, 2009). Shared profiles of exploration and attention may be built on an infant’s joint attention capacity, which emerges and strengthens across the second half of the first year (Mundy et al., 2007; Scaife & Bruner, 1975). In this period infants become increasingly sensitive to their interaction partner’s direction of gaze, leading to shared attention (Beier & Spelke, 2012). Shared emotional states with the caregivers may then be experienced frequently through daily face-to-face interactions with novel objects or events. The model by Creswell et al. (2010) suggests that this normative attention mechanism, may become maladaptive and mediate early anxiety transmission in cases where parental attention is repeatedly targeted to the threatening aspects of the environment. Biased attention in the parent may then trigger parental expressions of anxiety, fueling this cycle. An earlier study including parents with or without anxiety disorders noted that parental anxiety expressions towards novel stimuli, rather than the presence of an anxiety diagnosis determine child avoidant reactions to the stimuli (Aktar et al., 2013). Although this study did not measure infant and parent gaze to the novel stimuli, parents may induce an attention bias towards novel stimuli, coupled with a subsequent avoidant behavioral tendency in these situations. A longitudinal study in a sample of clinically and sub-clinically anxious parents and their infants that includes observations of parent-infant shared attention (in everyday interactions and computerized eye-tracking tasks), along with indices of parent and child anxiety dispositions would be the ideal follow-up design to gain further insight into shared attention to threat as a mechanism in anxiety transmission.

## Limitations and Future Directions

The following limitations should be considered while interpreting the current findings. First, it is important to note that only a few of the hypothesized associations were significant among all analyses conducted for the current study. Although the findings provided evidence for a significant association between parent and infant attention bias to threat in this community sample, the hypotheses regarding the role of attention in the hypothesized links between parent anxiety and infant temperament were, by large, not supported. Future research on these associations in community and clinical samples of parents with young children is needed for a more rigorous test of these hypothesized associations.

Second, we did not experimentally manipulate parental anxiety dispositions nor infant temperament. The associations between attention biases and anxiety dispositions were cross-sectional and correlational, precluding any prospective or causal inferences on the effect of anxiety dispositions on infant or parent attention to facial expressions. Recently, the importance of adopting a developmental approach was highlighted as crucial to understanding how normative attention biases in infancy may develop into a risk factor or mechanism for anxiety, and how infant and parent characteristics may play a role in this co-evolution (Burris et al., 2019; Field & Lester, 2010). Future studies should investigate these associations in longitudinal multi-method designs to establish a timeline, which is essential to understanding the developmental trajectories of attention biases.

Third, the current study did not include direct observations of parental anxiety or infant temperament. Parental anxiety symptoms and infant negative dispositions were only indirectly measured using questionnaires, which were completed by the parents. Future studies should consider measuring infant temperament and parental anxiety using naturalistic observations along with self-report measures. Fourth, the current analyses had to exclude a younger sub-sample due to difficulties related to eye-tracking, limiting the generalizations of our findings to younger ages. Finally, although parent emotion processing was incorporated in the current design, the eye-tracking data were only available from the specific parent who visited the lab, and the analyses of parental variables were limited to that parent. Future investigations should consider incorporating both parents, when available, for a more complete picture of the hypothesized associations within the family. Indeed, a family system approach often notes that the aggregated impact of the family on child development is more than a simple sum of individual contributions (Johnson & Ray, 2016). Despite these limitations, the current study constitutes the first evidence suggesting significant concordance in attention between parents and infants, prior to, or in the absence of extreme anxiety in the absence of extreme anxiety.

## Supplementary Information

Below is the link to the electronic supplementary material.

Supplementary file1 (DOCX 74 KB)

## Data Availability

All raw data and materials will be stored online within one month after publication to DataverseNL, available upon request from the corresponding author.

## Code Availability

All codes used during data processing will be stored online within one month after publication to DataverseNL, available upon request from the corresponding author.
