# Supplementary material for: Attention Biases to Threat in Infants and Parents: Links to Parental and Infant Anxiety Dispositions
Source: Res Child Adolesc Psychopathol. 2021 Sep 28;50(3):387–402. doi: 10.1007/s10802-021-00848-3 (PMC8885485; doi:10.1007/s10802-021-00848-3)
Supplement: Supplementary file 1 — Supplementary file1 (DOCX 74 KB) [file 10802_2021_848_MOESM1_ESM.docx]

| **Table S1**  *Initial multi-level regression of parents' dwell times on threat-relevant (versus happy) emotional expressions and parental anxiety dispositions (N=216)* | | | | | | | |
| --- | --- | --- | --- | --- | --- | --- | --- |
| **Fixed Effects** |  |  |  |  |  |  |  |
|  | *Numerator df* | *Denominator df* | *F* | *p* |  |  |  |
| Intercept | 1 | 215.97 | 0.43 | .513 |  |  |  |
| Emotion (threat-relevant versus happy) | 2 | 334.50 | 3.96 | .020 |  |  |  |
| Parent Gender (mother versus father) | 1 | 215.97 | 0.63 | .428 |  |  |  |
| Parental Anxiety Dispositions | 1 | 215.97 | 0.08 | .779 |  |  |  |
| Emotion * Parental Anxiety Dispositionss | 2 | 334.50 | 0.63 | .534 |  |  |  |
| Parent Gender * Parental Anxiety Dispositionss | 1 | 215.97 | 1.10 | .296 |  |  |  |
| Emotion * Parent Gender | 2 | 334.50 | 1.71 | .182 |  |  |  |
| Emotion * Parent Gender * Parental Anxiety Dispositions | 2 | 334.50 | 2.47 | .086 |  |  |  |
| **Estimates of Fixed Effects** |  |  |  |  |  |  |  |
| Parameters | *ß* | *SE* | *df* | *t* | *p* | 95% Confidence Intervals | |
|  |  |  |  |  |  | Lower  Bound | Upper Bound |
| Intercept | -.08 | .11 | 237.79 | -0.68 | .496 | -.30 | .15 |
| Angry versus Happy | .10 | .04 | 221.00 | 2.24 | .026 | .01 | .18 |
| Fearful versus Happy | .11 | .04 | 352.28 | 2.52 | .012 | .02 | .19 |
| Mother (vs. Father) | .16 | .13 | 237.79 | 1.17 | .244 | -.11 | .42 |
| Parental Anxiety Dispositions | -.10 | .12 | 237.79 | -0.85 | .399 | -.33 | .13 |
| Angry * Parental Anxiety Dispositions | -.03 | .05 | 221.00 | -0.57 | .570 | -.12 | .06 |
| Fearful * Parental Anxiety Dispositions | .06 | .04 | 352.28 | 1.30 | .195 | -.03 | .15 |
| Mother * Parental Anxiety Dispositions | .15 | .14 | 237.79 | 1.09 | .278 | -.12 | .42 |
| Mother * Angry | -.07 | .05 | 221.00 | -1.33 | .185 | -.17 | .03 |
| Mother * Fearful | -.09 | .05 | 352.28 | -1.79 | .075 | -.19 | .01 |
| Angry * Mother * Parental Anxiety Dispositions | .04 | .05 | 221.00 | 0.81 | .419 | -.06 | .15 |
| Fearful * Mother* Parental Anxiety Dispositions | -.07 | .05 | 352.28 | -1.36 | .174 | -.17 | .03 |
| **Estimates of Covariance Parameters** |  | *Estimate* | *SE* | *Wald Z* | *p* | *Lower*  *Bound* | *Upper Bound* |
| Repeated Measures | AR1 diagonal | .06 | .01 | 9.72 | <.001 | .05 | .07 |
|  | AR1 rho | .05 | .10 | 0.49 | .624 | -.15 | .25 |
| Intercept [subject = ID] | Variance | .75 | .07 | 10.09 | <.001 | .62 | .91 |
| *Notes.* *df=* degrees of freedom, *F* = F-value, *p* = significance level, *ß* = Beta, *SE* = Standard Error, *t* = t-value. | | | | |  |  |  |

| **Table S2**  *Initial multi-level regression of infant dwell times on threat-relevant (versus happy) emotional expressions, and infant anxiety dispositions (N=211)* | | | | | | | |
| --- | --- | --- | --- | --- | --- | --- | --- |
| **Fixed Effects** | *Numerator df* | *Denominator df* | *F* | *p* |  |  |  |
| Intercept | 1 | 210.95 | 0.10 | .750 |  |  |  |
| Age group (12 and 18 versus 6-month-olds) | 2 | 210.95 | 8.58 | <.001 |  |  |  |
| Emotion (threat-relevant versus happy) | 2 | 329.99 | 13.98 | <.001 |  |  |  |
| Infant Anxiety Dispositions | 1 | 210.95 | 0.25 | .618 |  |  |  |
| Emotion * Age group | 4 | 329.99 | 0.65 | .625 |  |  |  |
| Age group * Infant Anxiety Dispositions | 2 | 210.95 | 0.54 | .586 |  |  |  |
| Emotion * Infant Anxiety Dispositions | 2 | 329.99 | 0.24 | .787 |  |  |  |
| Emotion * Age group * Infant Anxiety Dispositions | 4 | 329.99 | 0.79 | .532 |  |  |  |
|  |  |  |  |  |  |  |  |
| **Estimates of Fixed Effects** |  |  |  |  |  |  |  |
| Parameter | *ß* | *SE* | *df* | *t* | *p* | 95% Confidence Intervals | |
|  |  |  |  |  |  | Lower  Bound | Upper Bound |
| Intercept | -.41 | .14 | 280.54 | -2.83 | .005 | -.69 | -.12 |
| 12 versus 6-month-olds | .53 | .18 | 280.54 | 2.87 | .004 | .17 | .89 |
| 18 versus 6-month-olds | .71 | .19 | 280.54 | 3.79 | <.001 | .34 | 1.07 |
| Angry versus Happy | -.16 | .09 | 210.12 | -1.76 | .080 | -.35 | .02 |
| Fearful versus Happy | .12 | .09 | 346.64 | 1.29 | .198 | -.06 | .30 |
| Infant Anxiety Dispositions | -.05 | .15 | 280.54 | -0.32 | .752 | -.33 | .24 |
| Angry * 12 versus 6-month-olds | .00 | .12 | 210.12 | 0.02 | .984 | -.23 | .24 |
| Angry * 18 versus 6-month-olds | .00 | .12 | 210.12 | 0.02 | .985 | -.24 | .24 |
| Fearful * 12 versus 6-month-olds | -.12 | .12 | 346.64 | -1.05 | .295 | -.35 | .11 |
| Fearful * 18 versus 6-month-olds | .01 | .12 | 346.64 | 0.10 | .922 | -.22 | .25 |
| 12 versus 6-month-olds * Infant Anxiety Dispositions | .10 | .19 | 280.54 | 0.55 | .583 | -.27 | .47 |
| 18 versus 6-month-olds * Infant Anxiety Dispositions | .10 | .18 | 280.54 | 0.52 | .605 | -.27 | .46 |
| Angry * Infant Anxiety Dispositions | -.06 | .09 | 210.12 | -0.63 | .527 | -.25 | .13 |
| Fearful * Infant Anxiety Dispositions | .01 | .09 | 346.64 | 0.09 | .931 | -.18 | .19 |
| Angry *12 versus 6-month-olds * Infant Anxiety Dispositions | .18 | .12 | 210.12 | 1.46 | .147 | -.06 | .42 |
| Angry *18 versus 6-month-olds * Infant Anxiety Dispositions | .10 | .12 | 210.12 | 0.81 | .418 | -.14 | .33 |
| Fearful *12 versus 6-month-olds * Infant Anxiety Dispositions | .05 | .12 | 346.64 | 0.44 | .660 | -.18 | .29 |
| Fearful * 18 versus 6-month-olds * Infant Anxiety Dispositions | -.05 | .12 | 346.64 | -0.42 | .673 | -.28 | .18 |
| **Estimates of Covariance Parameters** |  | *Estimate* | *SE* | *Wald Z* | *p* | *Lower*  *Bound* | *Upper Bound* |
| Repeated Measures | AR1 diagonal | .19 | .02 | 9.74 | <.001 | .15 | .23 |
|  | AR1 rho | .03 | .10 | 0.32 | .747 | -.17 | .23 |
| Intercept [subject = ID] | Variance | .69 | .08 | 9.18 | <.001 | .56 | .86 |
| *Notes.* *df=* degrees of freedom, *F* = F-value, *p* = significance level, *ß* = Beta, *SE* = Standard Error, *t* = t-value. | | | | |  |  |  |

| **Table S3**  *Initial linear regression of infant temperamental anxiety dispositions on parental anxiety dispositions (N= 196)* | | | | | | | |
| --- | --- | --- | --- | --- | --- | --- | --- |
|  |  |  |  |  |  |  |  |
|  |  |  |  |  |  |  |  |
|  | *B* | *SE* | *ß* | *t* | *p* | 95% Confidence Intervals | |
|  |  |  |  |  |  | Lower Bound | Upper Bound |
| Intercept | 2.27 | .11 |  | 21.30 | <.001 | 2.06 | 2.48 |
| 12 versus 6-month-olds | .38 | .14 | .32 | 2.68 | .008 | .10 | .67 |
| 18 versus 6-month-olds | .62 | .15 | .51 | 4.22 | <.001 | .33 | .91 |
| Parental Anxiety Dispositions | .32 | .35 | .13 | 0.93 | .353 | -.36 | 1.00 |
| Parental Anxiety Dispositions * 12 versus 6-month-olds | .42 | .44 | .14 | 0.96 | .340 | -.44 | 1.28 |
| Parental Anxiety Dispositions * 18 versus 6-month-olds | -.39 | .42 | -.14 | -0.92 | .358 | -1.23 | .45 |
| *Notes.* *ß = standardized beta,* *p* = significance level, *B* = unstandardized beta, *SE* = Standard Error, *t* = t-value. | | | | |  |  |  |

| **Table S4**  *Initial multi-level regression of infant dwell times on threat-relevant (versus happy) emotional expressions, age group, parent gender and parental dwell times (N =210)* | | | | | | | |
| --- | --- | --- | --- | --- | --- | --- | --- |
| **Fixed Effects** | *Numerator df* | *Denominator df* | *F* | *p* |  |  |  |
| Intercept | 1 | 216.40 | 0.36 | .548 |  |  |  |
| Parent Gender (mother versus father) | 1 | 216.54 | 1.28 | .259 |  |  |  |
| Age group (12 and 18 versus 6-month-olds) | 2 | 216.17 | 9.36 | <.001 |  |  |  |
| Emotion (threat-relevant versus happy) | 2 | 335.60 | 13.51 | <.001 |  |  |  |
| Parental Dwell Times | 1 | 550.42 | 1.64 | .200 |  |  |  |
| Age group * Parent Gender | 2 | 216.27 | 0.62 | .537 |  |  |  |
| Emotion * Parent Gender | 2 | 331.09 | 0.68 | .507 |  |  |  |
| Parent Gender * Parental Dwell Times | 1 | 547.61 | 0.96 | .328 |  |  |  |
| Emotion * Age group | 4 | 337.41 | 0.51 | .730 |  |  |  |
| Age group * Parental Dwell Times | 2 | 511.15 | 0.67 | .512 |  |  |  |
| Emotion * Parental Dwell Times | 2 | 353.95 | 0.13 | .876 |  |  |  |
| Emotion * Age group * Parent Gender | 4 | 333.32 | 1.19 | .313 |  |  |  |
| Age group * Parent Gender * Parental Dwell Times | 2 | 508.03 | 0.65 | .524 |  |  |  |
| Emotion * Parent Gender * Parental Dwell Times | 2 | 349.83 | 0.22 | .804 |  |  |  |
| Emotion * Age group * Parental Dwell Times | 4 | 348.27 | 0.47 | .757 |  |  |  |
|  |  |  |  |  |  |  |  |
| **Estimates of Fixed Effects** |  |  |  |  |  |  |  |
| Parameter | *ß* | *SE* | *df* | *t* | *p* | 95% Confidence Intervals | |
|  |  |  |  |  |  | Lower  Bound | Upper Bound |
| Intercept | -.24 | .18 | 284.03 | -1.33 | .183 | -.59 | .11 |
| Mother (vs. Father) | -.16 | .23 | 281.52 | -0.67 | .504 | -.61 | .30 |
| 12 versus 6-month-olds | .16 | .28 | 280.38 | 0.57 | .571 | -.39 | .71 |
| 18 versus 6-month-olds | .83 | .29 | 297.13 | 2.89 | .004 | .26 | 1.39 |
| Angry versus Happy | -.08 | .12 | 212.74 | -0.73 | .468 | -.31 | .14 |
| Fearful versus Happy | .14 | .11 | 346.46 | 1.27 | .205 | -.08 | .37 |
| Parental Dwell Times | .03 | .12 | 458.62 | 0.29 | .773 | -.20 | .27 |
| 12 versus 6-month-olds * Mother | .39 | .34 | 279.34 | 1.15 | .253 | -.28 | 1.07 |
| 18 versus 6-month-olds * Mother | -.23 | .35 | 292.23 | -0.66 | .508 | -.93 | .46 |
| Angry * Mother | -.08 | .15 | 211.96 | -0.55 | .584 | -.38 | .21 |
| Fearful * Mother | -.08 | .15 | 348.18 | -0.51 | .608 | -.37 | .22 |
| Mother * Parental Dwell Times | .09 | .16 | 434.93 | 0.54 | .587 | -.23 | .41 |
| Angry * 12 versus 6-month-olds | .17 | .18 | 213.52 | 0.96 | .338 | -.18 | .53 |
| Angry * 18 versus 6-month-olds | -.22 | .19 | 215.31 | -1.19 | .236 | -.59 | .15 |
| Fearful * 12 versus 6-month-olds | .12 | .18 | 344.40 | 0.69 | .488 | -.23 | .48 |
| Fearful *18 versus 6-month-olds | -.03 | .18 | 343.71 | -0.18 | .855 | -.40 | .33 |
| 12 versus 6-month-olds * Parental Dwell Times | .09 | .20 | 423.79 | 0.45 | .656 | -.31 | .49 |
| 18 versus 6-month-olds * Parental Dwell Times | -.22 | .26 | 625.83 | -0.88 | .381 | -.72 | .28 |
| Angry * Parental Dwell Times | .04 | *.08* | 214.40 | 0.55 | .580 | -.11 | .20 |
| Fearful * Parental Dwell Times | .09 | .08 | 346.85 | 1.18 | .239 | -.06 | .25 |
| Angry * 12 versus 6-month-olds * Mother | -.19 | .22 | 212.46 | -0.85 | .399 | -.62 | .25 |
| Angry * 18 versus 6-month-olds * Mother | .31 | .23 | 213.35 | 1.35 | .178 | -.14 | .75 |
| Fearful * 12 versus 6-month-olds* Mother | -.19 | .22 | 345.24 | -0.90 | .371 | -.62 | .23 |
| Fearful * 18 versus 6-month-olds* Mother | .11 | .22 | 345.49 | 0.48 | .629 | -.33 | .55 |
| 12 versus 6-month-olds * Mother * Parental Dwell Times | -.09 | .24 | 383.84 | -0.37 | .713 | -.56 | .39 |
| 18 versus 6-month-olds * Mother * Parental Dwell Times | .27 | .30 | 594.63 | 0.90 | .369 | -.32 | .87 |
| Angry * Mother * Parental Dwell Times | -.06 | .09 | 215.59 | -0.63 | .527 | -.24 | .12 |
| Fearful * Mother * Parental Dwell Times | -.05 | .09 | 344.97 | -0.49 | .627 | -.23 | .14 |
| Angry * 12 versus 6-month-olds * Parental Dwell Times | .05 | .10 | 214.46 | 0.52 | .604 | -.14 | .24 |
| Angry * 18 versus 6-month-olds * Parental Dwell Times | -.08 | .13 | 219.38 | -0.56 | .573 | -.34 | .19 |
| Fearful * 12 versus 6-month-olds * Parental Dwell Times | -.05 | .10 | 348.21 | -0.57 | .566 | -.24 | .13 |
| Fearful * 18 versus 6-month-olds * Parental Dwell Times | -.08 | .14 | 343.49 | -0.59 | .557 | -.35 | .19 |
| **Estimates of Covariance Parameters** |  | *Estimate* | *SE* | *Wald Z* | *P* | 95% Confidence Intervals | |
|  |  |  |  |  |  | *Lower*  *Bound* | *Upper Bound* |
| Repeated Measures | AR1 diagonal | .19 | .02 | 9.78 | <.001 | .15 | .23 |
|  | AR1 rho | .03 | .10 | .27 | .787 | -.18 | .23 |
| Intercept [subject = ID] | Variance | .70 | .08 | 9.17 | <.001 | .57 | .87 |
| *Notes.* *df=* degrees of freedom, *F* = F-value, *p* = significance level, *ß* = Beta, *SE* = Standard Error, *t* = t-value. | | | | |  |  |  |

| **Table S5**  *Initial multi-level regression of infant dwell times on threat-relevant (versus happy) emotional expressions, age group, and parental gender, dwell times and anxiety dispositions*  *(N =195)* | | | | | | | | | | | | |
| --- | --- | --- | --- | --- | --- | --- | --- | --- | --- | --- | --- | --- |
|  |  |  |  |  | |  | |  | |  | |  |
| **Fixed Effects** | *Numerator df* | *Denominator df* | *F* | *p* | |  | |  | |  | |  |
| Intercept | 1 | 251.04 | 0.67 | .415 | |  | |  | |  | |  |
| Parent Gender (mother versus father) | 1 | 249.93 | 2.46 | .118 | |  | |  | |  | |  |
| Age group (12 and 18 versus 6-month-olds) | 2 | 231.78 | 9.46 | <.001 | |  | |  | |  | |  |
| Emotion (threat-relevant versus happy) | 2 | 301.15 | 8.66 | <.001 | |  | |  | |  | |  |
| Parental Dwell Times | 1 | 580.96 | 1.34 | .247 | |  | |  | |  | |  |
| Parental Anxiety Dispositions | 1 | 204.04 | 0.01 | .938 | |  | |  | |  | |  |
| Age group * Parent Gender | 2 | 232.50 | 0.33 | .719 | |  | |  | |  | |  |
| Emotion * Parent Gender | 2 | 300.54 | 0.45 | .640 | |  | |  | |  | |  |
| Parent Gender * Parental Dwell Times | 1 | 578.89 | 0.93 | .336 | |  | |  | |  | |  |
| Parent Gender * Parental Anxiety Dispositions | 1 | 236.43 | 1.27 | .260 | |  | |  | |  | |  |
| Emotion * Age group | 4 | 301.66 | 0.69 | .602 | |  | |  | |  | |  |
| Age group * Parental Dwell Times | 2 | 554.92 | 1.22 | .296 | |  | |  | |  | |  |
| Age group * Parental Anxiety Dispositions | 2 | 230.10 | 1.05 | .353 | |  | |  | |  | |  |
| Emotion * Parental Dwell Times | 2 | 330.54 | 0.80 | .448 | |  | |  | |  | |  |
| Emotion * Parental Anxiety Dispositions | 2 | 306.36 | 0.04 | .957 | |  | |  | |  | |  |
| Parental Dwell Times * Parental Anxiety Dispositions | 1 | 440.74 | 0.19 | .660 | |  | |  | |  | |  |
| Emotion * Age group * Parent Gender | 4 | 301.66 | 1.69 | .153 | |  | |  | |  | |  |
| Age group * Parent Gender * Parental Dwell Times | 2 | 567.58 | 0.35 | .701 | |  | |  | |  | |  |
| Age group * Parent Gender * Parental Anxiety Dispositions | 2 | 219.08 | 0.26 | .769 | |  | |  | |  | |  |
| Emotion * Parent Gender * Parental Dwell Times | 2 | 332.94 | 1.51 | .223 | |  | |  | |  | |  |
| Emotion * Parent Gender * Parental Anxiety Dispositions | 2 | 310.66 | 1.42 | .242 | |  | |  | |  | |  |
| Parent Gender * Parental Dwell Times * Parental Anxiety Dispositions | 1 | 547.49 | 0.13 | .723 | |  | |  | |  | |  |
| Emotion * Age group * Parental Dwell Times | 4 | 322.61 | 0.08 | .989 | |  | |  | |  | |  |
| Emotion * Age group * Parental Anxiety Dispositions | 4 | 310.92 | 0.68 | .608 | |  | |  | |  | |  |
| Age group * Parental Dwell Times * Parental Anxiety Dispositions | 2 | 515.96 | 1.00 | .370 | |  | |  | |  | |  |
| Emotion* Parental Dwell Times * Parental Anxiety Dispositions | 2 | 390.98 | 1.03 | .357 | |  | |  | |  | |  |
|  |  |  |  |  | |  | |  | |  | |  |
| **Estimates of Fixed Effects** |  |  |  |  | |  | |  | |  | |  |
| Parameter | *ß* | *SE* | *df* | *t* | | *p* | | 95% Confidence Intervals | | | |  |
|  |  |  |  |  | |  | | Lower  Bound | | Upper Bound | |  |
| Intercept | -.23 | .21 | 258.64 | -1.08 | | .280 | | -.64 | | .18 | |  |
| Mother (vs. Father) | -.23 | .25 | 261.55 | -0.92 | | .357 | | -.73 | | .26 | |  |
| 12 versus 6-month-olds | .31 | .35 | 295.09 | 0.89 | | .376 | | -.37 | | .99 | |  |
| 18 versus 6-month-olds | .89 | .32 | 329.42 | 2.73 | | .007 | | .25 | | 1.52 | |  |
| Angry versus Happy | -.03 | .13 | 197.64 | -0.22 | | .826 | | -.29 | | .23 | |  |
| Fearful versus Happy | .18 | .13 | 326.48 | 1.38 | | .170 | | -.08 | | .43 | |  |
| Parental Dwell Times | .16 | .14 | 448.66 | 1.14 | | .255 | | -.12 | | .44 | |  |
| Parental Anxiety Dispositions | -.04 | .32 | 218.35 | -0.11 | | .909 | | -.66 | | .59 | |  |
| 12 versus 6-month-olds* Mother | .38 | .39 | 289.27 | 0.96 | | .336 | | -.40 | | 1.16 | |  |
| 18 versus 6-month-olds* Mother | -.24 | .38 | 308.50 | -0.63 | | .531 | | -.98 | | .51 | |  |
| Angry * Mother | -.18 | .16 | 196.44 | -1.10 | | .275 | | -.50 | | .14 | |  |
| Fearful * Mother | -.14 | .16 | 328.51 | -0.88 | | .381 | | -.45 | | .17 | |  |
| Mother * Parental Dwell Times | .16 | .19 | 448.32 | 0.85 | | .398 | | -.21 | | .54 | |  |
| Mother * Parental Anxiety Dispositions | .33 | .35 | 209.05 | 0.94 | | .349 | | -.36 | | 1.01 | |  |
| Angry * 12 versus 6-month-olds | .17 | .24 | 213.78 | 0.70 | | .482 | | -.30 | | .63 | |  |
| Angry * 18 versus 6-month-olds | -.34 | .22 | 212.03 | -1.58 | | .116 | | -.77 | | .09 | |  |
| Fearful * 12 versus 6-month-olds | .02 | .22 | 331.07 | 0.09 | | .931 | | -.42 | | .46 | |  |
| Fearful * 18 versus 6-month-olds | -.16 | .21 | 327.05 | -0.78 | | .434 | | -.58 | | .25 | |  |
| 12 versus 6-month-olds* Parental Dwell Times | -.19 | .40 | 561.89 | -0.49 | | .626 | | -.98 | | .59 | |  |
| 18 versus 6-month-olds* Parental Dwell Times | -.44 | .33 | 562.87 | -1.34 | | .182 | | -1.09 | | .21 | |  |
| 12 versus 6-month-olds* Parental Anxiety Dispositions | -.03 | .40 | 221.93 | -0.07 | | .944 | | -.82 | | .77 | |  |
| 18 versus 6-month-olds* Parental Anxiety Dispositions | -.33 | .36 | 221.82 | -0.93 | | .356 | | -1.04 | | .37 | |  |
| Angry * Parental Dwell Times | -.01 | .10 | 204.10 | -0.11 | | .910 | | -.21 | | .18 | |  |
| Fearful * Parental Dwell Times | .14 | .10 | 335.39 | 1.37 | | .171 | | -.06 | | .33 | |  |
| Angry * Parental Anxiety Dispositions | .13 | .13 | 198.19 | 0.94 | | .346 | | -.14 | | .39 | |  |
| Fearful * Parental Anxiety Dispositions | .09 | .13 | 331.68 | 0.70 | | .484 | | -.17 | | .35 | |  |
| Parental Dwell Times * Parental Anxiety Dispositions | .14 | .22 | 510.81 | 0.63 | | .528 | | -.30 | | .58 | |  |
| Angry * 12 versus 6-month-olds* Mother | -.18 | .27 | 211.94 | -0.67 | | .506 | | -.71 | | .35 | |  |
| Angry * 18 versus 6-month-olds* Mother | .47 | .25 | 205.78 | 1.88 | | .062 | | -.02 | | .97 | |  |
| Fearful * 12 versus 6-month-olds* Mother | -.12 | .26 | 330.75 | -0.45 | | .652 | | -.62 | | .39 | |  |
| Fearful * 18 versus 6-month-olds* Mother | .27 | .24 | 327.19 | 1.14 | | .257 | | -.20 | | .75 | |  |
| 12 versus 6-month-olds* Mother * Parental Dwell Times | -.02 | .44 | 545.29 | -0.05 | | .964 | | -.89 | | .85 | |  |
| 18 versus 6-month-olds* Mother * Parental Dwell Times | .28 | .36 | 581.30 | 0.78 | | .437 | | -.42 | | .98 | |  |
| 12 versus 6-month-olds* Mother * Parental Anxiety Dispositions | -.19 | .44 | 207.49 | -0.42 | | .674 | | -1.05 | | .68 | |  |
| 18 versus 6-month-olds* Mother * Parental Anxiety Dispositions | .07 | .41 | 222.70 | 0.16 | | .874 | | -.75 | | .88 | |  |
| Angry * Mother * Parental Dwell Times | -.03 | .13 | 211.93 | -0.21 | | .835 | | -.28 | | .23 | |  |
| Fearful * Mother * Parental Dwell Times | -.21 | .13 | 330.45 | -1.59 | | .113 | | -.47 | | .05 | |  |
| Angry * Mother * Parental Anxiety Dispositions | -.19 | .11 | 200.77 | -1.69 | | .093 | | -.41 | | .03 | |  |
| Fearful * Mother * Parental Anxiety Dispositions | -.10 | .11 | 338.94 | -0.90 | | .368 | | -.31 | | .12 | |  |
| Mother * Parental Dwell Times * Parental Anxiety Dispositions | .07 | .20 | 547.49 | 0.36 | | .723 | | -.33 | | .48 | |  |
| Angry * 12 versus 6-month-olds* Parental Dwell Times | .04 | .13 | 204.02 | 0.34 | | .733 | | -.21 | | .29 | |  |
| Angry * 18 versus 6-month-olds* Parental Dwell Times | .02 | .16 | 211.86 | 0.13 | | .895 | | -.29 | | .33 | |  |
| Fearful * 12 versus 6-month-olds* Parental Dwell Times | .05 | .13 | 328.85 | 0.38 | | .703 | | -.20 | | .30 | |  |
| Fearful * 18 versus 6-month-olds* Parental Dwell Times | .07 | .16 | 327.53 | 0.44 | | .659 | | -.24 | | .38 | |  |
| Angry * 12 versus 6-month-olds* Parental Anxiety Dispositions | -.05 | .13 | 199.67 | -0.40 | | .692 | | -.30 | | .20 | |  |
| Angry * 18 versus 6-month-olds* Parental Anxiety Dispositions | .00 | .12 | 198.92 | 0.01 | | .996 | | -.24 | | .24 | |  |
| Fearful * 12 versus 6-month-olds* Parental Anxiety Dispositions | -.14 | .12 | 327.54 | -1.12 | | .265 | | -.37 | | .10 | |  |
| Fearful * 18 versus 6-month-olds* Parental Anxiety Dispositions | .02 | .12 | 334.44 | 0.20 | | .842 | | -.21 | | .26 | |  |
| 12 versus 6-month-olds* Parental Dwell Times * Parental Anxiety Dispositions | .01 | .18 | 454.32 | 0.07 | | .941 | | -.34 | | .37 | |  |
| 18 versus 6-month-olds* Parental Dwell Times * Parental Anxiety Dispositions | -.27 | .22 | 536.52 | -1.25 | | .214 | | -.71 | | .16 | |  |
| Angry * Parental Dwell Times * Parental Anxiety Dispositions | -.06 | .06 | 233.52 | -0.95 | | .342 | | -.19 | | .07 | |  |
| Fearful * Parental Dwell Times * Parental Anxiety Dispositions | -.10 | .07 | 343.78 | -1.40 | | .162 | | -.23 | | .04 | |  |
| **Estimates of Covariance Parameters** |  | *Estimate* | *SE* | *Wald Z* | | *p* | | 95% Confidence Intervals | | | |  |
|  |  |  |  |  | |  | |  | |  | |  |
|  |  |  |  |  | |  | | *Lower*  *Bound* | | *Upper Bound* | |  |
| Repeated Measures | AR1 diagonal | .19 | .02 | 8.90 | | <.001 | | .15 | | .23 | |  |
|  | AR1 rho | .06 | .11 | 0.56 | | .576 | | -.16 | | .27 | |  |
| Intercept [subject = ID] | Variance | .60 | .07 | 8.52 | | <.001 | | .47 | | .75 | |  |
| *Notes.* *df=* degrees of freedom, *F* = F-value, *p* = significance level, *ß* = Beta, *SE* = Standard Error, *t* = t-value. | | | | |  | |  | |  | |  | |
